# Reply to Ronsivalle, S.; Di Luca, M. Exploring the Choice of Graft Materials in Tympanoplasty: A Perspective on the Use of Temporalis Fascia and Cartilage Grafts. Comment on “Ferlito et al. Type 1 Tympanoplasty Outcomes between Cartilage and Temporal Fascia Grafts: A Long-Term Retrospective. *J. Clin. Med*. 2022, *11*, 7000”

**DOI:** 10.3390/jcm13041169

**Published:** 2024-02-19

**Authors:** Antonino Maniaci, Jerome R. Lechien, Miguel Mayo-Yanez, Mario Lentini, Federico Merlino

**Affiliations:** 1 Department of Medicine and Surgery, School of Medicine, University of Enna “Kore”, 94100 Enna, Italy; 2 Department of Anatomy and Experimental Oncology, Mons School of Medicine, UMONS Research Institute for Health Sciences and Technology, 7000 Mons, Belgium; jerome.lechien@umons.ac.be; 3 Otorhinolaryngology—Head and Neck Surgery Department, Complexo Hospitalario Universitario A Coruña (CHUAC), 15006 A Coruña, Spain; miguelmmy@gmail.com; 4 ASP Ragusa—Hospital Giovanni Paolo II, 97100 Ragusa, Italy; marlentini@tiscali.it; 5 Department of Medical and Surgical Sciences and Advanced Technologies “GF Ingrassia”, ENT Section, University of Catania, Via Santa Sofia, 95100 Catania, Italy; federico.merlino93@gmail.com

We sincerely appreciate the valuable insights Ronsivalle et al. have provided in their analysis [1] of our recent research on the use of temporalis fascia and cartilage grafts in type I tympanoplasty [2]. Your thoughtful and engaging discussion regarding the findings of our study emphasizes the significance and relevance of this field of research. Your astute observation regarding the potential impact of graft thickness on auditory outcomes is well taken. While our study encompassed various factors for analysis, a comprehensive investigation specifically focused on the influence of graft thickness was beyond the scope of the paper. However, we agree with your suggestion that a more concentrated study in this area could yield significant insights, potentially enhancing surgical outcomes.

Furthermore, we acknowledge the potential influence of patient age on the tensile strength of fascia and cartilage materials and its implications for graft durability [3,4]. Although our study emphasized the long-term stability of cartilage grafts, we recognize the importance of discussing age-related changes in fascia integrity in greater detail. Such considerations could assist in graft selection for older patients [5]. The primary objective of our study was to provide comparative data to aid in the decision-making process between temporalis fascia and cartilage grafts in tympanoplasty. We appreciate your recognition of our efforts in this regard. As you aptly pointed out, this decision is complex and necessitates a delicate balance between graft integrity and acoustic performance tailored to each patient’s unique circumstances [6]. The standardized surgical approach, as described, did not specify whether additional procedures were conducted during the operation. Such procedures might have included canalplasty to enhance exposure or the “pull-back” technique to anchor the anterosuperior fascia. Since the influential report by Salen et al. [7] and Jansen et al. [8] on employing the cartilage-perichondrial composite graft for tympanic membrane reconstruction, several adaptations have emerged. In addition, the introduction of the “Pac Man” graft, a modification to inlay tympanoplasty using fascial and cartilage materials, represents an innovative advancement in surgical techniques. This adaptation could potentially lead to a reduction in the incidence of unfavorable outcomes and may simplify the handling of the graft during surgery, particularly in certain challenging cases. Your insightful analysis and suggestions will undoubtedly shape our future research endeavors. We are grateful for your engagement and eagerly anticipate further dialogue as we strive to enhance patient outcomes and satisfaction in tympanoplasty procedures.
